# Manifestation of an undifferentiated uterine sarcoma in a 51 years old patient and its prognosis: A case report

**DOI:** 10.1097/MD.0000000000032552

**Published:** 2022-12-30

**Authors:** Miloš Pantelić, Ljiljana Gvozdenovic, Milana Panjković, Marko Stojić, Dragan Stajić, Aleksandra Petrić, Sonja Pop Trajković, Milan Trenkić, Dušan Simić, Lazar Živadinović, Aleksandar Živadinović

**Affiliations:** a Faculty of Medicine, University of Novi Sad, Novi Sad, Serbia; b University Clinical Center of Vojvodina, Clinic for Obstetrics and Gynecology, Novi Sad, Serbia; c University Clinical Center of Vojvodina, Clinic for Anesthesia, Intensive Therapy and Pain Therapy, Novi Sad, Serbia; d University Clinical Center of Vojvodina, Department of Pathology and Histology, Novi Sad, Serbia; e Faculty of Medicine University of Niš, Niš, Serbia; f University Clinical Center Niš, Clinic for Gynecology and Obstetrics, Niš, Serbia; g Health Center Niš, Womens Health Protection Office, Niš, Serbia.

**Keywords:** prognosis, ultrasonography, undifferentiated uterine sarcoma, uterine sarcoma

## Abstract

Undifferentiated uterine sarcoma is a rare histological subtype of uterine sarcoma. This study aimed to summarize the clinical and pathological presentation of this case.

**Case report:**

A 51-years-old patient was admitted to the clinic because of severe pain in the lower abdomen, and scanty bleeding from the genitals. Gynecological examination revealed an enlarged uterus. Conventional and Doppler transvaginal sonography detected a tumorously altered uterus with a maximum diameter of 20 cm a tumefaction with unclear borders and a diameter of 10 cm, with hyperechoic and hypoechoic fields within the tumefaction, presenting pathological vascularization and reduced values of the (Pulsatile index  ≤ 1) and (Resistance index  ≤ 0.40). Preoperatively, the chest, abdomen, and pelvis were examined. The patient underwent surgery and total abdominal hysterectomy with bilateral salpingo-oophorectomy, and partial omentectomy, with complete removal of the tumor. A pathohistological diagnosis, of undifferentiated uterine sarcoma, was made by excluding other types of uterine sarcomas. At the control examination after completion of chemotherapy, recurrence was ascertained.

**Conclusion:**

undifferentiated uterine sarcoma is an aggressive malignant tumor that in most cases shows rapid progression of the disease after complete resection of the tumor, with a poor prognosis.

## 1. Introduction

Uterine sarcomas are rare malignant tumors that arise from the mesenchymal tissues of the uterus, that is, the stroma of the endometrium, uterine muscles and connective tissue. They make up 1% of female genital tract malignancies and 3% to 7% of all uterine malignancies.^[1]^ Understanding stromal endometrial sarcomas has significantly evolved and led to new classifications of stromal endometrial sarcomas.^[2]^ Changes in their classifications have contributed to insufficient knowledge, regarding risk factors, therapeutic modalities and prognosis. Undifferentiated uterine sarcoma is a term adopted according to the world health organization classification to replace previously used undifferentiated endometrial sarcoma. The world health organization (2014) classified the main types of uterine sarcomas into: carcinosarcomas (malignant mesodermal mixed tumors, account for 50% of cases), leiomyosarcomas (30%), endometrial stromal sarcomas (15%) and undifferentiated uterine sarcomas (5%).^[3]^ Undifferentiated uterine sarcomas are a rare and aggressive type of tumor, of heterogeneous biology, associated with a very poor prognosis.^[4]^

In this study, we present the case of undifferentiated uterine sarcoma and the course of the disease.

## 2. Case report

A 51-years-old woman, was admitted to the gynecology and obstetrics clinic due to severe pain in the lower abdomen, weakness, malaise, small amount of postmenopausal bleeding, difficulty with urination and stool lasting for several weeks.

Upon admission to the Clinic, the polymerase chain reaction test for SARS-CoV-2 virus was negative.

Patient was 3 years menopausal and had a history of myoma for 11 years. Eight months earlier, a fractionated exploratory curettage was performed, which did not indicate the existence of a malignant tumor.

On bimanual pelvic examination, the vagina was elastic, passing for 2 transverse fingers, the cervix was shortened with closed cervical canal and the uterus was enlarged, firm and limited mobile, adnexal without palpable tumefactions. Conventional transvaginal sonography revealed a completely altered uterus with a maximum diameter of 20 cm, with undifferentiated cavum and tumefactation on the anterior wall of the uterus of unclear borders, with hyper and hypoechoic fields within the tumefact, about 10 cm in size. The adnexa were bilaterally without pathological tumufeactions, both ovaries were of normal size. Douglas space was without free fluid. Color Doppler tranvaginal sonography revealed pathological vascularization, that is, highly pronounced and numerous blood vessels with irregular and chaotic branching (without lumen arrangement) with present arterial-venous shunts. Pulse Doppler indicated markedly reduced values of the (Pulsatily index  ≤ 1) and the (Resistance index  ≤ 0.40). The described changes in vascularization were especially emphasized within the tumefact.

Preoperatively, the chest, abdomen and pelvis were imaged, without suspected tumefications outside the uterus.

The patient underwent surgery and a total abdominal hysterectomy with bilateral salpingo-oophorectomy, adhesiolysis and partial omentectomy was performed. Tumor tissue was removed completely, without residual tumor tissue in the abdominal cavity. Intraoperatively, the uterus was about 20 cm in diameter in adhesions with sigmoid colon, left adnexa and Douglas space. A small amount of free abdominal fluid was aspirated and sent for cytological analysis. The uterus had an uneven surface, which was held intact only by a thin serosa without a clear ruptured site. During the release of the appendages, there was a spontaneous rupture of the left lateral wall of the uterus and hemorrhagic and necrotic contents came out of the uterus, bizarre in appearance, partly gelatinous.

**Figure 1.**
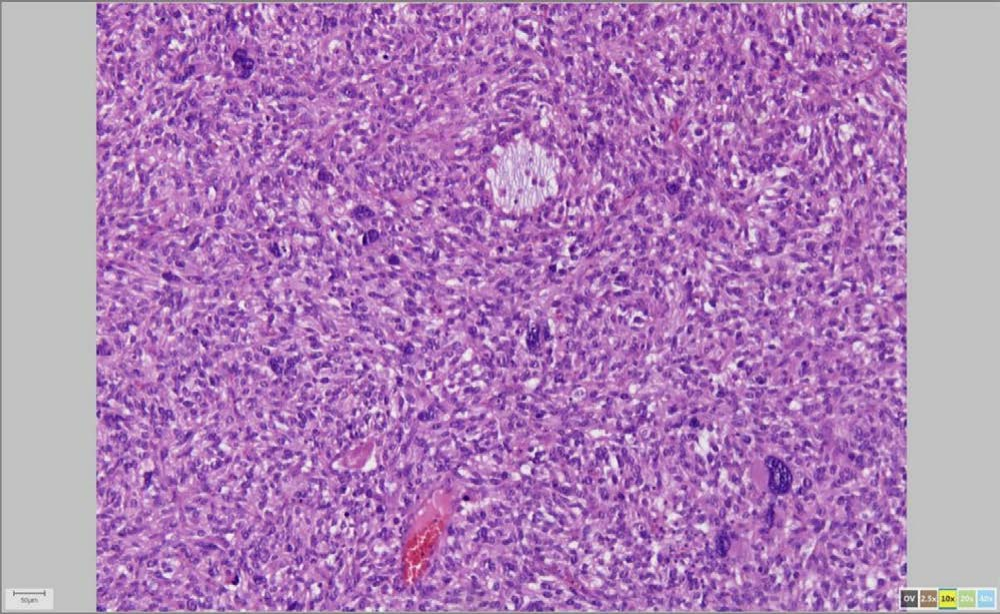
UUS Histological pattern, HEx 10.jpg. UUS = undifferentiated uterine sarcoma.

Cytological examination of abdominal fluid revealed numerous erythrocytes and small number of mesothelial cells without finding of atypical cells. Macroscopically, the body of the uterus was enlarged, 20 × 14 × 12 cm in diameter, with some adhesions on the surface. On cross-section of the uterine body, the cavum was dislocated and inconspicuous due to the presence of a clearly demarcated intramural nodule, yellow to pink in color and soft in consistency on the cut surface, 8 cm in largest diameter. Along with the described tumor nodule, softed areas were also observed in the myometrium. Samples were taken on several occasions for pathohistological examination, embedded in paraffin blocks and stained by the standard H&E method (Fig. 1).

Microscopically, the tumor tissue was composed of atypical spindle and round cells of medium size. Focally, atypical cells were pleomorphic with bizarre nuclei and number of pathological mitosis was increased. Tumor cells were arranged in a storiform and nesting arrangement with presence of tumor cells in numerous blood vessels. The stroma was sparse with inflammatory infiltrate and rare foci of myxoid degeneration. Larger areas of necrosis and bleeding were found in the tumor. Tumor tissue invaded all layers of the uterine wall with serosal infiltration as well as infiltration of the left adnexa.

**Figure 2.**
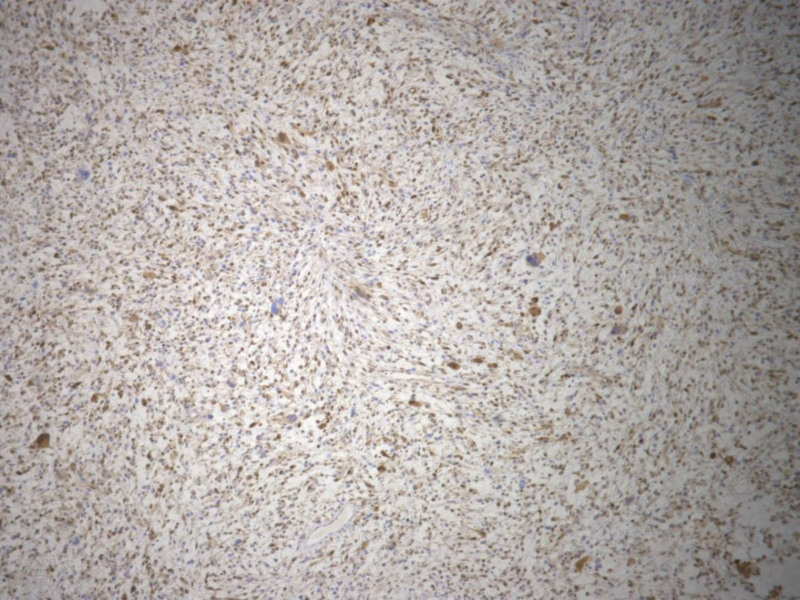
UUS Cyclin D1 staining of tumor cells,x 5.jpg. UUS = undifferentiated uterine sarcoma.

Immunohistochemical examination was performed to establish diagnosis. Tumor cells were negative for SMA, Desmine, S-100, Caldesmon, CD34, AE1/AE3, CD31, ERG, GLUT, D2-40, CD68, CD117, p16 and progesterone receptors while CD 10, estrogene receptors, Cyclin D1, DOG, p53 were positive in tumor cells. Ki 67 proliferative index was about 80% (Fig. 2).

According to the histological and immunohistochemical analysis, leiomyosarcoma was excluded. Major differential diagnostic problem was High-grade endometrial sarcoma and although molecular analysis was not performed, immunohistochemical results strongly support the definitive diagnosis of Undifferentiated sarcoma of uterine body.^[5]^ According to the international federation of gynecologists and obstetricians classification, it is stage II A of disease with lymphovascular invasion present. According to the indication of the oncology commission, the patient received 3 cycles of polychemotherapy postoperatively, according to the protocol, including Doxorubicin and Ifosfamide, at the Oncology Institute of Vojvodina. At the control examination after the prescribed therapy, there was a progressive recurrence in the left half of the pelvis (from vaginal cuff with involvement of the iliac region; vascular invasion; infiltration of the left ureter) with the appearance of constant pain. Teleradiotherapy was prescribed.

## 3. Discussion

The incidence of uterine sarcoma in the population ranges from 1.55 to 1.95 per 1,00,000 women per year. It is estimated that 0.1% to 0.3% of patients operated on for presumed uterine leiomyoma have uterine sarcoma.^[6–8]^ Patients with undifferentiated uterine sarcoma (USS) are mostly postmenopausal, with an average age of about 60 years. Abnormal uterine bleeding is the most common. The etiology is unknown. In postmeopausal patients who experience severe lower abdominal pain, genital bleeding, signs of acute abdomen, uterine rupture caused by uterine sarcoma involving all layers of the uterine wall should be considered. In our study, there was a threatening uterine rupture that manifested during the operation during the release from the surrounding structures. Risk factors are ethnicity, history of pelvic radiation, history of tamoxifen treatment, rapid growth of myometrial lesions in menopausal patients.^[3,4,9]^

Undifferentiated uterine sarcomas histologically show myometrial invasion, severe nuclear pleomorphism, high mitotic activity and/ or tumor cell necrosis, and without a specific line of differentiation show alternative morphological and immunohistochemical characteristics compared to endometrial sarcomas. Pathohistological diagnosis is made by excluding other types of uterine sarcoma.^[5,10]^ In 60% of patients, the disease is diagnosed in an advanced stage of the disease (stages III and IV) with a very poor prognosis (survival less than 2 years).^[11,12]^

Uterine sarcomas have similar clinical characteristics to benign leiomyomas. Ultrasound findings of a large myometrial tumor of the uterus, complex structure (different echogenicity, internal irregular cystic areas), with frequent pathological vascularization accompanied by gynecological symptoms (especially abnormal vaginal bleeding) and postmenopausal status of the patient indicate malignancy.^[8]^ Tumor stage is the most important prognostic factor.^[3,13]^ Surgery is considered the basis of treatment, while other treatments, such as chemotherapy or radiation therapy, have shown limited results. Surgical treatment involves hysterectomy with bilateral salpingo-oophorectomy. Complete cytoreduction should be the goal of surgery. The role of lymphadenectomy remains controversial. When the disease is limited to the uterus, systemic pelvic and paraaortic lymphadenectomy is not recommended. Indication for removal of enlarged lymph nodes is disseminated or recurrent disease.

Chemotherapy may be considered when there is a risk of hematogenous spread and distant metastases. The benefits of chemotherapy for patients who have undergone complete resection of the disease limited to the uterus are also controversial. The rarity of these tumors makes it difficult to develop and evaluate new therapies.

The presence of estrogen and progesterone positive tumors opens the possibility of hormone therapy in the treatment of undifferentiated uterine sarcomas. Metastatic symptoms as well as local disease status can be treated with radiotherapy. Persistent and recurrent disease has a similar behavior of soft tissue sarcoma with a poor prognosis.^[3,10,11,14,15]^

## 4. Conclusion

Undifferentiated uterine sarcoma is a rare and aggressive tumor, with a poor prognosis. Significance before and postoperative imaging of the chest, abdomen and pelvis is due to the choice of adequate treatment after surgical resection. Further development of molecular biology is aimed at better knowledge of these tumors, progress in diagnostics, which would achieve a better therapeutic protocol and improve survival.

## Author contributions

**Conceptualization:** Ljiljana Gvozdenovic, Milana Panjković, Marko Stojić, Dragan Stajić, Aleksandra Petrić, Sonja Pop Trajković, Milan Trenkić, Dušan Simić, Lazar Živadinović, Aleksandar Živadinović.

**Data curation:** Miloš Pantelić, Ljiljana Gvozdenovic, Milana Panjković, Marko Stojić, Dragan Stajić.

**Formal analysis:** Miloš Pantelić, Ljiljana Gvozdenovic, Aleksandra Petrić, Sonja Pop Trajković, Milan Trenkić, Dušan Simić, Lazar Živadinović, Aleksandar Živadinović.
